# Analysis of Morphological Parameters to Differentiate Rupture Status in Anterior Communicating Artery Aneurysms

**DOI:** 10.1371/journal.pone.0079635

**Published:** 2013-11-13

**Authors:** Ning Lin, Allen Ho, Nareerat Charoenvimolphan, Kai U. Frerichs, Arthur L. Day, Rose Du

**Affiliations:** 1 Department of Neurosurgery, Brigham and Women’s Hospital, Boston, Massachusetts, United States of America; 2 Harvard Medical School, Boston, Massachusetts, United States of America; 3 Department of Neurosurgery, University of Texas Medical School at Houston, Houston, Texas, United States of America; Children's National Medical Center, Washington, United States of America

## Abstract

In contrast to size, the association of morphological characteristics of intracranial aneurysms with rupture has not been established in a systematic manner. We present an analysis of the morphological variables that are associated with rupture in anterior communicating artery aneurysms to determine site-specific risk variables. One hundred and twenty-four anterior communicating artery aneurysms were treated in a single institution from 2005 to 2010, and CT angiograms (CTAs) or rotational angiography from 79 patients (42 ruptured, 37 unruptured) were analyzed. Vascular imaging was evaluated with 3D Slicer© to generate models of the aneurysms and surrounding vasculature. Morphological parameters were examined using univariate and multivariate analysis and included aneurysm volume, aspect ratio, size ratio, distance to bifurcation, aneurysm angle, vessel angle, flow angle, and parent-daughter angle. Multivariate logistic regression revealed that size ratio, flow angle, and parent-daughter angle were associated with aneurysm rupture after adjustment for age, sex, smoking history, and other clinical risk factors. Simple morphological parameters such as size ratio, flow angle, and parent-daughter angle may thus aid in the evaluation of rupture risk of anterior communicating artery aneurysms.

## Introduction

The prevalence of cerebral aneurysms is estimated to be 1–3% [1], [2], [3] and unruptured aneurysms appear to be identified with increasing frequency [3], [4]. The optimal management of an incidentally discovered cerebral aneurysm remains a controversial topic in neurosurgery, as the potential risk of aneurysm rupture must be balanced with the risks of therapeutic options such as microsurgical clipping or endovascular coiling. As a result of the International Study of Unruptured Intracranial Aneurysms (ISUIA) [5], [6], [7], [8], treatment decision of unruptured intracranial aneurysms (UIAs) is based mainly on the size and location of the aneurysm in the anterior or posterior circulation. A recent Japanese study (UCAS) further categorized the dependence of rupture risk on specific locations [9]. Additionally, analyzing morphological characteristics of an aneurysm has been increasingly used to evaluate its rupture risk. A number of geometric parameters, including aspect ratio [10], [11], size ratio [12], [13], aneurysm inflow angle [14], and volume-to-ostium ratio [15], have been shown to have greater association with aneurysm rupture status than size alone. However, such studies grouped together all aneurysm types, which may confound characteristics that are specific to the vascular anatomy associated with a specific location. We previously reported that greater aspect ratio, larger aneurysm flow angle, and smaller parent-daughter angle indicated higher risk of rupture for middle cerebral artery aneurysms [16]. Others have also reported anatomical risk factors that are specific to aneurysm location [17], [18]. Herein, we present a large sample of anterior communicating artery (ACoA) aneurysms that were assessed using a diverse array of morphological variables to determine the parameters associated with aneurysm rupture at this specific location.

## Methods

### Ethics Statement

The study was approved by the Brigham and Women’s Hospital Institutional Review Board. Written consent from the patients was waived by the Institutional Review Board.

### Patient Selection

The study population consisted of 124 consecutive patients with anterior communicating artery (AcoA) aneurysms that were treated at the Brigham and Women’s Hospital between 2005 and 2010. We included aneurysms arising from the A1–A2 junction in the cohort but excluded distal ACA aneurysms. Re-operated aneurysms, fusiform aneurysms, or those associated with arteriovenous malformations were also excluded from the study, resulting in 118 patients. Pre-operative CTAs were not available or of poor quality in 39 aneurysms, leaving 79 aneurysms (42 ruptured, 37 unruptured) available for analysis. Medical records were reviewed to obtain demographic and clinical information, including patient data on vascular risk factors such as smoking status, family history, presence of multiple aneurysms, history of hypertension, and prior history of SAH.

### Reconstruction of 3D Models

Three-dimensional (3D) models of ACoA aneurysms were generated from pre-operative CT angiography (CTA) using 3D Slicer© software. Detailed methods of constructing and refining the 3D models have been described previously [16]. All CTAs were performed on a Siemens® SOMATOM Definition scanner with slice thickness of 0.75 mm and increment of 0.5 mm. All measurements were obtained independently by two observers, and the average value was used for subsequent statistical analyses.

### Definition of Morphological Parameters

**Figure 1:**
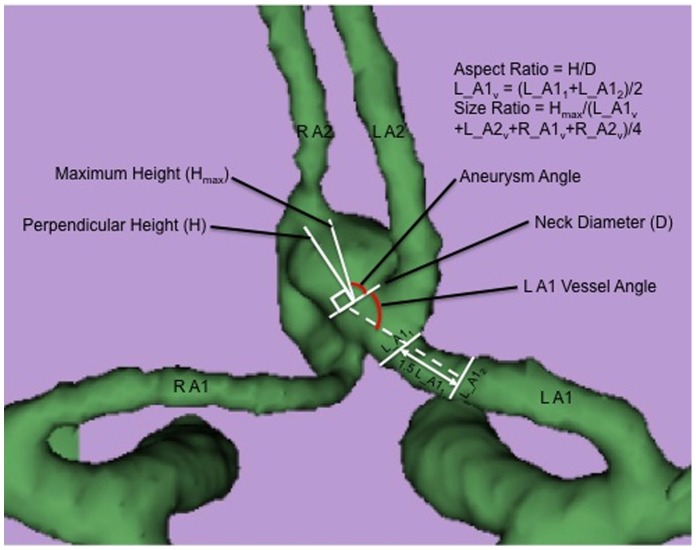
Definition of morphological parameters used in the analysis. Maximum aneurysm height (H_max_) is measured between the center of the aneurysm neck and the greatest distance to the aneurysm dome. Maximal perpendicular height (H) is the largest perpendicular distance from the neck of the aneurysm to the dome of the aneurysm. Aspect ratio is calculated as the ratio of the H and neck diameter of the aneurysm. Size ratio is the ratio between H_max_ and the mean vessel diameter of all branches associated with the aneurysm (L_A1_v_, L_A2_v_, R_A1_v_, R_A2_v_). Aneurysm angle is the angle formed between the neck of the aneurysm and the maximum height of the aneurysm (H_max_). Vessel angle is the angle between the parent vessel and the plane of the aneurysm neck. Flow angle is the angle between the maximum height of the aneurysm and the parent vessel.

**Figure 2:**
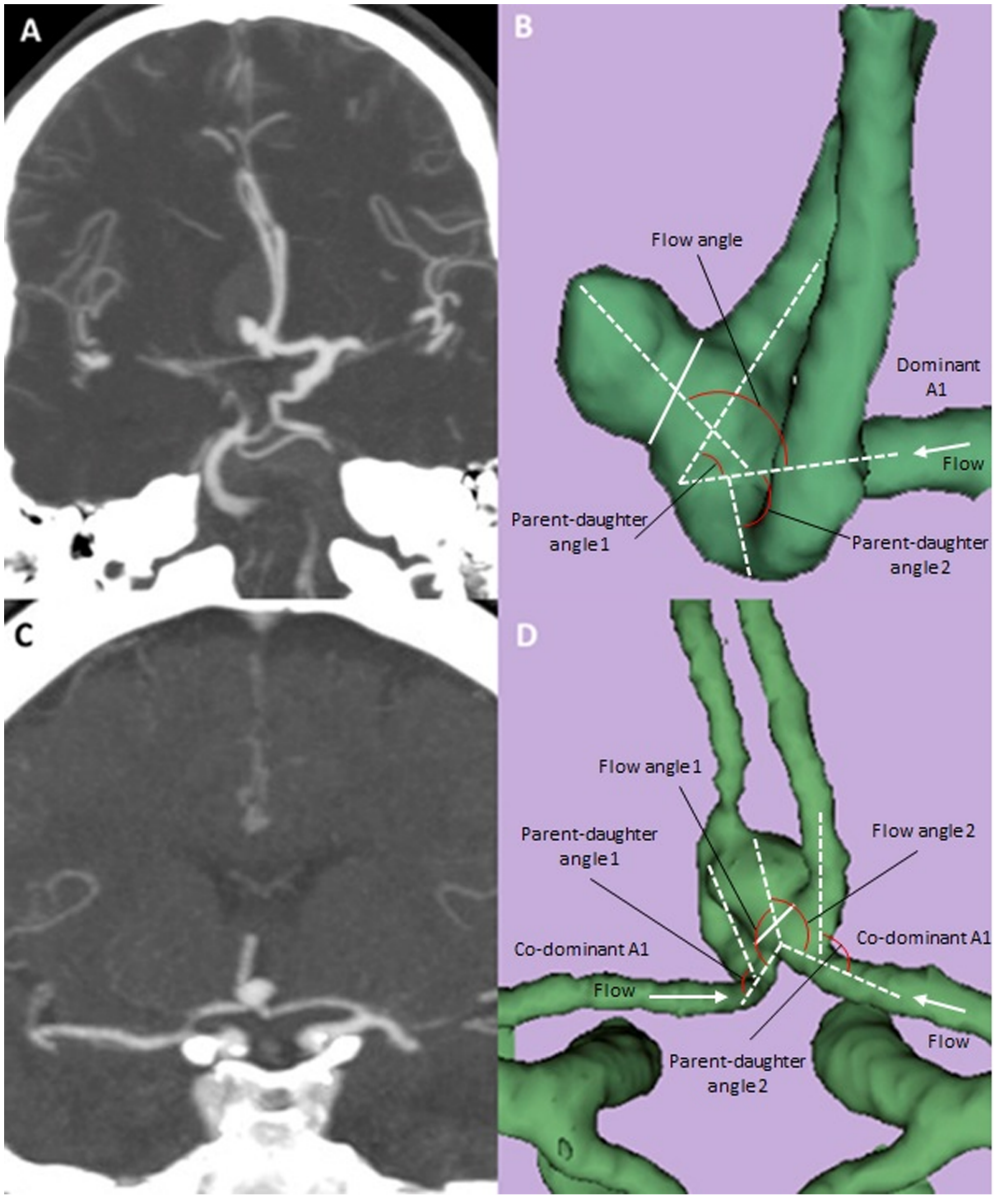
Computed Tomography Angiogram (CTA) and three-dimensional models of anterior communicating aneurysms with a dominant A1 or with co-dominant A1’s. Coronal MIP images are shown for an ruptured AcoA aneurysm with a dominant left A1 (A) and an unruptured AcoA aneurysm with co-dominant A1’s (C). The corresponding 3-D reconstructed images from Slicer are shown in B and D with definitions of morphological parameters. Solid line (B and D) indicates aneurysm neck.

Eight morphological parameters were examined in 3D aneurysm models: aneurysm diameter, aneurysm volume, aspect ratio, size ratio, aneurysm angle, vessel angle, flow angle, and parent-daughter angle. These parameters have been defined previously [16] and are described briefly below (Figures 1 and 2). Aneurysm diameter is the largest cross-sectional diameter. Maximum aneurysm height (H_max_ in Figure 1) is measured between the center of the aneurysm neck and the greatest distance to the aneurysm dome. Maximal perpendicular height (H in Figure 1) is the largest perpendicular distance from the neck of the aneurysm to the dome of the aneurysm. Aspect ratio is the ratio of the maximum perpendicular height of the aneurysm to the average neck diameter of the aneurysm. Size ratio is the ratio between the maximum aneurysm height and the mean vessel diameter of all branches associated with the aneurysm. The vessel diameter of a particular branch (e.g. L_A1_v_ in Figure 1) is determined by averaging the diameter of the cross-section of this vessel just proximal to the neck of the aneurysm (L_A1_1_) and the diameter of the cross section at 1.5xL_A1_1_ from the neck of the aneurysm (L_A1_2_). Aneurysm angle is the angle formed between the neck of the aneurysm and the maximum height of the aneurysm. Vessel angle is the angle between the parent vessel and the plane of the aneurysm neck. Flow angle is the angle between the maximum height of the aneurysm and the parent vessel. Parent-daughter angle is the angle between the parent vessel and the daughter vessel(s). If more than one daughter vessel is present, the angle associated with each daughter vessel is averaged to calculate a composite parent-daughter angle.

Of note, the measurement of flow angle and parent-daughter angle was achieved differently based on whether there were co-dominant A1 arteries or one A1 was dominant/obligate (Figure 2). If one A1 artery was dominant or obligate, the flow angle was formed between the vector of maximum height of the aneurysm, and the vector of centerline of the dominant A1, and the parent-daughter angle was calculated to be the average of the two angles formed by the dominant A1 and two A2 arteries (Figure 2B). On the other hand, if the A1 arteries were co-dominant, both were considered parent vessels and the flow angle was calculated as the average of each A1 and the aneurysm projection, and the parent-daughter angle was calculated to be the average of the angle between each A1 and the ipsilateral A2 artery (Figure 2D).

### Statistical Analysis

SAS version 9.2 (SAS Institute Inc., Cary, North Carolina) and Excel 2007 (Microsoft Corp., Redmond, WA) were used for all statistical analyses. Differences in demographic and clinical characteristics by rupture status were examined using chi-square and two-tailed t-tests for binary and continuous variables, respectively. Univariate analysis was performed to compare the value of each morphological parameter between the ruptured and unruptured groups. Multivariate logistic regression was used to calculate the odds ratios (OR) and 95% confidence intervals (CI) for the likelihood of aneurysm rupture after adjusting for age, sex, smoking status, family history, presence of multiple aneurysms, hypertension, and prior history of SAH. Statistical significance was defined as a type I error of less than 0.05.

## Results

Demographic and clinical information of the study population is listed in Table 1. The mean age was 53.3 (±11.7) years, and 50 patients were female (63.3%). Patients with ruptured aneurysms were more likely to have a history of smoking than those with unruptured aneurysms (57.1% vs. 29.7%), whereas those with unruptured AcoA aneurysms were more likely to harbor multiple aneurysms (Table 1).

**Table 1:** Demographic information and clinical risk factors for patients with ruptured and unruptured anterior communicating artery (ACoA) aneurysms.

	Unruptured	Ruptured	*p* value
N	37	42	
Mean age (SD)	54.6 (13.1)	52.1 (11.9)	0.57
Female (%)	26 (70.3%)	24 (57.1%)	0.09
Hypertension (%)	17 (45.9%)	20 (47.6%)	0.48
Smoking (%)	11 (29.7)	24 (57.1)	0.05
Multiple aneurysms (%)	11 (29.7%)	5 (11.9%)	0.04
Family history (%)	4 (10.8%)	6 (14.3%)	0.79
Prior SAH (%)	2 (5.4%)	1 (2.4%)	0.45

P value indicates the level of statistical significance that the value of certain variable differs between the ruptured group and unruptured group (p<0.05 is considered statistically significant). SAH, subarachnoid hemorrhage.

When the sizes of A1 arteries were considered, 25 aneurysms had co-dominant A1’s and 54 had a dominant or obligate A1. We used univariate analysis to examine pre-defined morphological parameters individually and compared their values between the ruptured and unruptured groups (Table 2). Ruptured aneurysms were associated with larger aneurysm angle (95.5 vs. 84.4, p = 0.05) and flow angle (125.7 vs. 112.6, p = 0.05), smaller parent-daughter angle (68.2 vs. 80.3, p = 0.04), as well as higher size ratio (3.22 vs. 2.34, p = 0.03) and aspect ratio (1.39 vs. 1.07, p = 0.04). Aneurysm size, as estimated by either the maximal cross-sectional diameter or the aneurysm volume, did not significantly differ between the ruptured and unruptured groups. In a multivariate logistic regression model adjusted for both morphological and clinical risk factors, aspect ratio, aneurysm angle, flow angle, size ratio, and parent-daughter angle were evaluated as independent variables and the results were summarized in Table 3. The analysis showed that greater size ratio (OR = 1.28, 95% CI = 1.15–1.76, p = 0.03), larger flow angle (OR = 1.05, 95% CI = 1.004–1.11, p = 0.04), and smaller parent-daughter angle (OR = 0.95, 95% CI = 0.91–0.99, p = 0.04) were associated with aneurysm rupture, whereas aneurysm angle (p = 0.10) and aspect ratio (p = 0.26) were no longer statistically significant parameters (Table 3).

**Table 2:** Univariate analyses for the morphological parameters measured for ruptured and unruptured ACoA aneurysms.

	Unruptured	Ruptured	Odds Ratio	*p* value
	mean (SD)	mean (SD)	(95% CI)	
N	37	42		
Dominant A1				
Left	9 (24.3%)	20 (47.6%)	2.82 (0.90–8.62)	
Right	14 (37.8%)	11 (26.2%)	1.00 (0.33–3.05)	0.09
Dominant	14 (37.8%)	11 (26.2%)	1 [Reference]	
Maximal Diameter (mm)	5.66 (3.49)	6.08 (2.78)	0.99 (0.82–1.20)	0.60
Aneurysm volume (mm^3^)	131.7 (141.7)	139.1 (167.9)	0.99 (0.99–1.02)	0.86
Aspect Ratio	1.07 (0.51)	1.39 (0.68)	2.24 (1.04–5.93)	0.04
Aneurysm Angle	84.4 (15.2)	95.5 (24.2)	1.03 (1.00–1.06)	0.05
Size ratio	2.34 (1.23)	3.22 (1.73)	1.58 (1.03–2.68)	0.03
Flow angle	112.6 (17.8)	125.7 (16.8)	1.08 (1.02–1.15)	0.05
Parent-daughter angle	80.3 (17.2)	68.2 (24.1)	0.97 (0.94–1.00)	0.04

P value and odds ratios indicate the level of statistical significance that the value of certain variable differs between the ruptured group and unruptured group (p<0.05 is considered statistically significant).

**Table 3:** Multivariate analysis after adjustment for clinical and morphological risk factors.

	Odds Ratio (95% CI)	*p* value
Aspect Ratio	3.97 (0.91–17.2)	0.26
Size ratio	1.28 (1.15–1.76)	0.03
Parent-daughter angle	0.95 (0.91–0.99)	0.04
Flow angle	1.05 (1.004–1.11)	0.04
Aneurysm Angle	1.02 (0.95–1.06)	0.10

A multivariate logistic regression model was constructed to ascertain morphological parameters that were significant predictors of aneurysm rupture, after adjusting for demographic and clinical risk factors. P value and odds ratios indicate the level of statistical significance for certain parameter in the multivariable regression model (p<0.05 is considered statistically significant).

**Figure 3:**
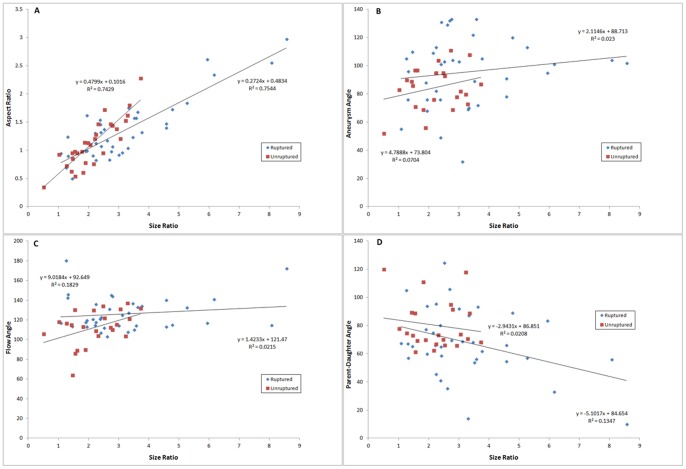
Scatter plots depicting the interaction between size ratio and other morphological parameters. Linear regression analysis for scatter plot of size ratio and aspect ratio yielded an R^2^ value of 0.75 for the ruptured group and 0.74 for the unruptured group (A), suggesting inter-dependency of the two variables. No clear dependency was observed between size ratio and aneurysm angle (B), flow angle (C), or parent-daughter angle (D).

**Figure 4:**
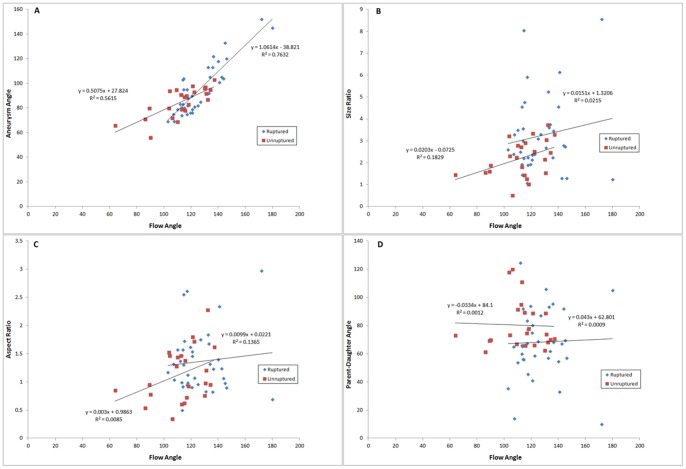
Scatter plots depicting the interaction between flow angle and other morphological parameters. Linear regression analysis for scatter plot of aneurysm angle and flow angle yielded an R^2^ value of 0.76 and 0.56 for the ruptured and unruptured groups, respectively (Figure A). No apparent relationship was found between flow angle and size ratio (B), aspect ratio (C), or parent-daughter angle (D).

To investigate inter-dependency of the morphological parameters, we used 2D-scatterplots to analyze the interactions among the morphological parameters that were different between the ruptured and unruptured aneurysms in univariate analysis. Size ratio (Figure 3) and flow angle (Figure 4) were utilized as independent variables, while aspect ratio, aneurysm angle, and parent-daughter angle were plotted as dependent variables. Size ratio could be linearly related to aspect ratio, and a simple linear regression analysis yielded an R^2^ value of 0.75 for the ruptured group and 0.74 for the unruptured group (Figure 3A). No apparent dependency was observed between size ratio and aneurysm angle (R^2^ = 0.023 for ruptured group, 0.0704 for unruptured group; Figure 3B), size ratio and flow angle (R^2^ = 0.0215 for ruptured group, 0.183 for unruptured group; Figure 3C), or size ratio and parent-daughter angle (R^2^ = 0.135 for ruptured group, 0.0208 for unruptured group; Figure 3D). Similarly, a linear relationship existed between flow angle and aneurysm angle, with the R^2^ value of 0.76 and 0.56 for the ruptured and unruptured groups, respectively (Figure 4A), whereas no clear relationship was found between flow angle and size ratio, aspect ratio, or parent-daughter angle (Figure 4B, 4C, and 4D).

## Discussion

The rupture risk of a cerebral aneurysm depends on a number of demographic and biological factors in addition to the size and location of the aneurysm [1], [5], [19]. The effect of aneurysm morphology and hemodynamic profile on the risk of rupture has received increasing attention because it has long been thought that “dangerous” aneurysms with high rupture risks could be distinguished from “safe” ones based on geometric properties. A list of recent publications on this topic has been summarized in Table 4. The parameters evaluated in these studies have largely fallen into two categories: morphological features measured from CTA or digital subtracted angiography (DSA), and hemodynamic features measured from computational fluid dynamics (CFD) models. For example, Raghavan et al. evaluated 8 geometric factors in 27 aneurysms (9 ruptured, 18 unruptured), and found that ruptured aneurysms were associated with significantly higher aspect ratio, undulation index, and nonsphericity index [11]. Dhar et al. [13] examined 45 aneurysms (20 ruptured, 25 ruptured), and reported that size ratio was the most significant predictor of aneurysm rupture. We previously presented a group of 79 MCA aneurysms and found that ruptured aneurysms had greater aspect ratio, larger flow angle, and smaller parent-daughter angle than the unruptured ones [16]. In this study, we focused on simple morphological parameters that can be easily measured in the clinical setting from CTAs or angiograms.

**Table 4:** Morphological and hemodynamic parameters evaluated in the literature.

Study	Year	Number ofaneurysms	Location ofaneurysms	Parameters evaluated	Significant parameters
Raghavan [11]	2005	57	All	Morphological parameters	Undulation index, aspect ratio, ellipticity index,nonsphericity index, mean curvature norm
Cebral [28]	2005	62	All	Hemodynamic parameters	Impingement region, jet size, stability of flow pattern
Dhar [13]	2008	45	All	Morphological parameters	Size ratio, undulation index, aneurysm angle
Castro [25]	2009	26	AcoA	Hemodynamic parameters	Maximum wall shear stress
Xiang [21]	2011	119	All	Morphological andhemodynamic parameters	Size ratio, wall shear stress, oscillatory shear index
Cebral [20]	2011	210	All	Hemodynamic parameters	Maximum wall shear stress, shear concentration,viscous dissipation ratio
Chien [29]	2011	50	All	Morphological parameters	Aneurysm surface to bounding sphere surface
Lin [16]	2012	79	MCA	Morphological parameters	Aspect ratio, flow angle, parent-daughter angle
Baharoglu [23]	2012	271	All	Morphological andHemodynamic parameters	Size ratio, inflow angle, height/width ratio
Matsukawa [24]	2013	140	AcoA	Morphological parameters	Anterior projection, presence of a bleb, aneurysm size >5 mm
Current study		79	AcoA	Morphological parameters	Size ratio, flow angle, parent-daughter angle

Additionally, computational fluid dynamic (CFD) models can be constructed through invasive and non-invasive vascular imaging to estimate hemodynamic stress on vessel walls and its relationship to aneurysm rupture; however, the CFD results have been conflicting with regards to wall shear stress. Cebral et al. analyzed a large cohort of 210 intracranial aneurysms using image-based CFD under different flow conditions. The authors reported that ruptured aneurysms were associated with larger maximum wall shear stress, higher shear concentration, and lower viscous dissipation ratio [20]. Xiang et al. analyzed 3-D angiographic images and CFD models of 119 aneurysms and studied both morphological and hemodynamic parameters. They found that larger size ratio, lower average wall shear stress, and higher oscillatory shear index (that is, the disturbance of the flow field) were associated with aneurysm rupture [21]. Miura et al. studied 106 MCA aneurysms and also found that lower wall shear stress was associated with rupture status [22]. In a recent study of 271 aneurysms, Baharoglu et al. argued that the hemodynamic profiles in a side-wall aneurysm and a bifurcation aneurysm might be completely different so that interpretations of quantitative CDF analyses could yield conflicting results depending on the type of aneurysm [23]. Our approach in examining morphological parameters in a location specific manner may therefore yield results that would otherwise have been confounded by the heterogeneity of the samples, as has been the case in prior studies.

The vascular anatomy and hemodynamics in the region of anterior communicating artery is notably more complex than in other areas of the intracranial circulation [24], [25], [26] due to the relative dominance of A1 arteries, configuration of A2 arteries, and different locations along the anterior communicating complex where an aneurysm could arise. For A1-dominant and A1-obligate aneurysms, we considered only the dominant/obligate A1 artery as the parent artery, as it has been demonstrated that the non-dominant A1 usually did not provide flow into the aneurysm [25]. A number of studies aimed to investigate morphological features of AcoA aneurysms and hemodynamics of the anterior communicating complex specifically. Castro et al. analyzed 18 ruptured and 8 unruptured ACoA aneurysms with patient-specific computational fluid dynamics models and found that ruptured aneurysms were associated with small impaction zones, higher inflows, and greater mean wall shear stress [25]. The relationship between wall shear stress and rupture status, however, remains controversial as other investigators including Fukazawa et al. [27] and Miura et al. [22] reported that aneurysm rupture point usually was within areas of low wall shear stress. Matsukawa et al. studied morphological parameters in 140 consecutive ACoA aneurysms, and reported that the presence of blebs, aneurysm size >5 mm, and anterior dome pointing were associated with aneurysm rupture [24].

In our series of 79 anterior communicating artery aneurysms we found size ratio, flow angle, and parent-daughter angle to be highly associated with aneurysm rupture in a multivariate logistic analysis. These results were obtained from a cohort of aneurysms in a largely homogeneous location (ACoA or A1/A2 junction) with adjustment of patients’ demographic and clinical risk factors. These findings are mostly consistent with our own study of a large cohort of MCA aneurysms [16] and reaffirmed that geometric parameters of an intracranial aneurysm, measured from invasive or non-invasive vascular imaging, would usually describe one of three distinct aspects of the aneurysm hemodynamics: the morphology of the aneurysm itself, the interaction between the aneurysm and the associated parent and daughter vessels, and the relationship among the surrounding vasculature.

With respect to parameters that are intrinsic to the aneurysm, aspect ratio was found to be highly associated with MCA aneurysm rupture [16], but was not a significant factor in multivariable analyses for the ACoA aneurysms, whereas size ratio was. This is likely because aspect ratio and size ratio are correlated and hence not independent parameters. The relationship between the aneurysm and surround vasculature as characterized by the flow angle can also account for the projection of aneurysm dome. The projection of the aneurysm dome was likely related to the flow angle formed by A1 and the aneurysm dome. Analyzing values of flow angle based on aneurysm dome directions demonstrated that anteriorly-pointing aneurysms have the largest flow angle, consistent with results from Matsukawa et al [18], although it was not statistically significant. Finally, the relationship among the surrounding vasculature is accounted for by the parent-daughter angle. We had first investigated the parent-daughter angle in MCA aneurysms. Smaller parent-daughter angle was associated with MCA aneurysm rupture [16], and also with ACoA aneurysm rupture in the current study. This parameter describes the angio-architecture of the vessels surrounding the aneurysm and thus is independent of the aneurysm itself. Additional work is necessary to ascertain how the size of parent-daughter angle changes the hemodynamic stress profile of the vessel walls and affects the rupture risk of an aneurysm.

A number of limitations exist for our study and should be taken into consideration while interpreting these results. It is a retrospective analysis of patients with AcoA aneurysms who were treated either surgically or endovascularly; those with unruptured aneurysms who were followed conservatively were not included in the study. Thus, the study population is biased toward morphologically complex and “dangerous” aneurysms. Moreover, surface irregularities, such as daughter domes or blebs, were not considered during our analyses. Nevertheless, excluding such surface parameters ensured that morphological analyses were less affected by the process of rupture. Size ratio, flow angle, and parent-daughter angle were unlikely to be significantly altered after aneurysm rupture compared with the unruptured state.

## Conclusion

Morphological parameters such as size ratio, flow angle, and parent-daughter angle were highly associated with anterior communicating artery aneurysm rupture. These simple morphological parameters may be used in conjunction with aneurysm size, location, as well as other demographic and clinical risk factors to aid in the prediction of the rupture risk of anterior communicating artery aneurysms.
